# A Clinical Lecture on Cancer of the Pylorus

**Published:** 1883-01

**Authors:** Thomas F. Rochester

**Affiliations:** Professor of the Principles and Practice of Medicine, University of Buffalo


### A Clinical Lecture on Cancer of the Pylorus.

By Thomas F. Rochester, M. D.,
Professor of the Principles and Practice of Medicine, University of Buffalo.

Reported by John H. Pryor, M. D.,
Resident Physician, Buffalo General Hospital.

Gentlemen—The patient before you presents the appearance of one who has long been the victim of chronic disease. He is greatly emaciated, looks wan and feeble, and it is probable that there is considerable disturbance of assimilation. His face wears a weary look, and the lines there, doubtless, have been traced by worry and continuous pain. Although very anæmic, yet a hectic flush is upon his cheek. The eye is sunken and heavy; the mouth has lost its firmness and relaxed into weakness; all expression of hope and determination is gone, and there appears the picture of resignation, calm endurance and indifference. It is the typical countenance of one who has felt torturing pain and struggled through sleepless nights. You cannot fail to notice the marks of advanced life, which indicate that old age has laid her benumbing hand upon this man, to soften his pain by subduing his strength.

Not long ago I described this face to you, and now my words have taken form. Some of you may have noted that his cough, while I am speaking, is not associated with much effort, seems a matter of slight annoyance, while he expectorates easily. Upon looking at the sputum, I find it to be muco-purulent in character and distinctly the expectoration of chronic bronchitis. Later on, we will listen to his chest for the peculiar sounds which are characteristic of this disease.

We will now hear his history. His voice is feeble; fearing that its tones will not be audible to all, I will repeat the words after him. He is seventy-two years of age; has no hereditary taint; always enjoyed good health until recently. Six weeks ago he first complained of indigestion. At first there was loss of appetite, pain after eating and heart-burn; then later on, he cannot remember the date, he commenced to vomit. About five weeks ago he vomited a considerable quantity of blood, since which time he has retained nothing on the stomach and does not care to eat, because his food gives him pain and is so soon regurgitated. At the commencement of this attack he had diarrhœa, but of late has been obstinately constipated; has shooting, lancinating pains over the epigastrium, and that is all. The history being incomplete, I shall endeavor to draw out a fuller and more exact report by a few direct questions. It is very evident that all of these symptoms point to the food tract, or organs of digestion.

But we will not allow a preconceived notion to so influence our examination as to tinge the diagnosis. If you do not consult this rule, you will find yourself subjecting every answer to the judgment of this premature opinion, thus leading you into an inexcusable blunder. From his replies I learn that the most annoying symptom of indigestion has been and is now acidity, with sour eructations; the vomiting has been persistent and occurs directly after meals; the food is rejected, undigested and unchanged. He does not vomit at other times. The blood which he vomited was fluid and red; the ingestion of food is followed by intense and lasting pain, not localized, but radiating and extending into the hypochondriac region; the pain is constant and is only intensified after eating; has no fever, nor does he complain of anything else.

We will next turn our attention to the physical signs which may be present: The tongue has a brown fur and is somewhat dry; placing my finger upon the wrist, I perceive a tortuous, atheromatous artery, with a slow and feeble pulse. Again we are reminded of old age. I promised to verify the diagnosis of chronic bronchitis. With my ear against the chest, I hear sibilant and sonorous rales everywhere, and some subcrepitant rales; these, combined with the expectoration and age, settle all question in regard to that matter.

An inspection of the abdomen shows little, save flattening of its walls, but feeling carefully, from place to place, I find a resisting, firm tumor at the region of the pyloric orifice, where there should be resiliency. When I percuss over it, a dull sound is emitted, and you know that it is normally tympanitic. Continuing to percuss lightly, I map out a circumscribed area of dullness about as large as a small orange, while he winces and tells me that it is very tender. Normally the pyloric orifice is located at this point, midway between the right nipple and umbilicus, while the cardiac orifice is higher up, at the junction of the sixth costal cartilage with the sternum, a little to the left. It is well to be careful in your statement, when speaking of the position of the stomach, because it is very changeable, and especially so in disease. I have seen the lower edge of the stomach as low as the pubes, again, the upper edge as high as the second rib. In organic disease at the pylorus, the stomach is liable to undergo hypertrophy with dilatation, for the food collecting in the stomach, having no exit, sometimes remains for days, with great distension of this viscus. Not unfrequently disease of the liver and pancreas simulate organic disease at the pylorus. But a study of the symptoms helps one to differentiate. Having carried in mind the history elicited, the diagnosis begins to assume the color of cancer. Let us, then, gather these symptoms into a cluster, subject them to an analysis, and see what they tell.

They confine us to chronic catarrh, gastric ulcer, and cancer. Dyspepsia is common to all, perhaps more especially marked in cancer and catarrh. Acidity is generally greater in cancer, while vomiting is a constant symptom. In such a case the food may be retained for a day or two. Often the time of vomiting indicates the seat of disease. If at the cardiac orifice, the food is almost immediately regurgitated. I once saw a case, however, where the œsophagus had become enlarged into a pouch and contained a large quantity of food. If at the pyloric orifice, the rule is variable. Sometimes the food remains for days and is then vomited in large amount. Hemorrhage usually occurs at the commencement and toward the close of the disease. The character of the pain in cancer is somewhat distinctive; it is constant, sharp, cutting and radiating.

In catarrh, vomiting is not so frequent, and the food ejected is frequently mixed with stringy mucus. Again, a watery secretion is often vomited during fasting, especially when complicated by congestion of the liver, kidney or spleen. Hemorrhage is rare. The pain is intermittent, and of a dull, aching kind. The vomiting in ulcer occurs at different times after meals, dependent on the seat of the ulcer. It is not persistent, and is frequently accompanied with slight hæmatemesis. The blood is usually red and liquid, but may resemble coffee grounds. There is localized pain, which is described as sharp, burning and acute. It is greatly increased on entrance of food, and relieved after vomiting. The epigastric region is tender in all, but in ulcer it is often confined to one spot.

I do not think it necessary to continue in this way any further. I have narrated to you the principal symptoms associated with these diseases with a view to their differentiation. If you have followed me closely, and remember the distinctive signs which I have pointed out to you, it must be very plain that this man is suffering from cancer of the stomach. It yet remains, however, for us to decide upon its seat. As to the variety of cancer we can only conjecture. Statistics prove that cancer affecting this organ is of the scirrhus variety in the large proportion of cases. The location of the disease is surely at the pylorus. The presence of the hard lump in this region, and the fact that the food passes into the stomach, rather preclude consideration of the cardiac orifice. To be sure that fluids pass beyond the cardiac orifice, it is sometimes well to ask the patient to drink, and applying the stethoscope to the epigastric region you can hear the fluid chink into the viscus. The prognosis, as a necessity, must be unfavorable. It is death, usually, at the end of a year. The treatment is only palliative. The existence of pain calls for the free exhibition of anodynes. It is often necessary to add lime water to milk and administer pepsin and bismuth in connection. No matter if they do vomit, some little is always retained. Occasionally you may resort to a trick in dietetics, beautifully described by William Hunter in "Watson's Practice." Feed them a teaspoonful often, and gradually increase the amount from day to day. Of course rectal alimentation is to be considered. During the closing days it often becomes the only source of supplying nutriment. Of late years a new operation (gastrotomy) has come into practice, but it has not justified itself, nor proved that it warrants general adoption. So far the results have not brought benefit sufficiently great to compensate for the suffering and extreme illness consequent upon its performance. Before leaving this subject, I would speak to you of a symptom which may present itself during the last few days of the disease and is apt to be misleading. I refer to the cessation of vomiting and the retention of food. This is due to a degenerative change assisted by ulceration at the pyloric orifice. The cancerous mass breaks down, and a small opening is produced by the ulcerative process which allows a certain amount of food passage into the intestine. I have noticed this condition several times at the autopsy.

